# Assessing the Welfare of Spiny Lobsters and True Lobsters in Aquaria: Biology-Informed Best-Practice Guidelines for Captive Conditions

**DOI:** 10.3390/ani15162397

**Published:** 2025-08-15

**Authors:** Lorenzo Fruscella, Robert W. Elwood, Annamaria Passantino

**Affiliations:** 1 School of Design, University of Greenwich, Park Row, London SE10 9LS, UK; 2 Animal Law Italia (NGO), Via Rocco Dicillo 1, 70131 Bari, Italy; l.fruscella@ali.ong; 3 School of Biological Sciences, Queen’s University, Belfast BT9 5DL, UK; r.elwood@qub.ac.uk; 4 Department of Veterinary Sciences, University of Messina, Via Palatucci Annunziata, 98168 Messina, Italy

**Keywords:** lobster, welfare, aquaria, spiny, guidelines, claws, behaviour, tanks, light, shelter

## Abstract

The welfare of decapod crustaceans held in captivity, whether in aquariums, holding facilities, seafood restaurants, supermarkets, or fishmongers, is attracting growing attention and concern. This review focuses on three species that are commonly found in European commercial environments: the European spiny lobster (*Palinurus elephas*), the European lobster (*Homarus gammarus*), and the American lobster (*Homarus americanus*). We examine the biology and behavioural ecology of these decapods, emphasising their natural habits and environmental requirements. Based on current scientific knowledge, we propose conditions for their confinement that better align with their biological and behavioural needs. These crustaceans are commonly subjected to stressful captive environments characterised by overcrowding, claw immobilisation through banding, an absence of shelter or hiding spaces, and continuous exposure to direct lighting. Such practices contrast starkly with the animals’ innate behaviours and natural habitats, likely compromising their physical health and psychological welfare. It is thereby recommended that spiny lobsters and lobsters be kept separated and stocked at an appropriate density, that each animal should be provided with a shelter and have enough space to turn around, that true lobsters should never have their claws tied, that no direct light should be placed above the aquaria, and that water quality should be routinely monitored. By exploring the welfare challenges associated with confining these species, this review aims to promote a more ethical and humane approach to their care in commercial settings, and to encourage practices that enhance their quality of life in captivity.

## Simple Summary

The welfare of decapod crustaceans kept in tanks in holding facilities, restaurants, supermarkets, and fishmongers is of increasing concern. We focus on three commonly kept species in Europe, namely the European spiny lobster (*Palinurus elephas*), the European lobster (*Homarus gammarus*), and the American lobster (*Homarus americanus*). This review considers the biology of these animals, taking into account their natural behaviour and needs, to then suggest suitable captive conditions based on scientific evidence. In general, these animals are exposed to high stocking densities, claw banding, lack of shelters, and direct light, and these are likely to negatively impact their welfare. These captive conditions contrast with the nature of these animals, and provisions should be made to align the conditions of the holding environment with their ethological needs. By addressing the welfare concerns associated with the captive maintenance of these decapods, this review aims to contribute to their ethical treatment in captivity and promote their welfare in commercial settings.

## 1. Introduction

Crustaceans are a diverse subphylum of the phylum Arthropoda that inhabit marine, freshwater, and terrestrial ecosystems. In Europe, commercially significant species, primarily from the order Decapoda, include spiny lobsters, true lobsters, crabs, and various prawn and shrimp species. These decapods are commonly kept in aquaria within holding facilities, restaurants, fishmongers, and supermarkets, where they are often kept live with the justification that it maintains their “freshness”. Historically, little attention was given to the welfare of these animals due to the belief that they lacked the capacity to feel pain, reacting solely through nociceptive reflexes to harmful stimuli. Nociceptive reflexes are short-term responses to a stimulus that are not influenced by other motivational requirements and do not include long-term behavioural changes, such as avoidance learning or attending to the affected area. However, recent studies have shown that many responses of decapods to noxious stimuli are not merely reflexive but suggest the ability to experience pain [1,2,3,4,5]. These studies support the view that decapods possess sensory mechanisms that detect harmful stimuli and engage in complex behavioural responses that are consistent with the idea of sentience. For example, Elwood [1,2] describes motivational trade-offs (e.g., hermit crabs abandoning low-quality shells when shocked), prolonged grooming of affected areas, and swift avoidance learning, which are all indicators of central processing rather than simple reflexes. Rowe [3] emphasises the need for ethical review of decapod research, citing evidence of protective behaviours and learned avoidance as evidence of pain experience. Conte et al. [4] report physiological stress responses (e.g., elevated lactate) and persistent distress behaviour following noxious stimuli. Crump et al. [5] further conclude that decapods meet multiple criteria for sentience, including nociception, learning capacity, and behavioural flexibility. Together, this growing body of evidence supports the need for welfare-focused practices in the handling and housing of decapods.

A notable report commissioned by the UK government from the London School of Economics and Political Science (LSE) further recognised decapods and cephalopods as having a high probability of being sentient with the ability to experience a range of emotions, including pain and distress [6]. This resulted in a change to UK law that recognised decapods as sentient. Despite this recognition, the conditions under which these animals are held in aquaria often fail to align with their natural behavioural needs. Crustaceans such as lobsters are solitary and territorial and require shelters for protection from light, but they are often housed in environments lacking these essential conditions. They are also typically kept at high stocking densities, leading to numerous welfare issues such as stress, disease, and aggression [7,8,9,10].

There is very limited species-specific information available for the majority of decapods displayed in aquaria, such as crabs. For these animals, there is a lack of research regarding suitable stocking densities, aggression, shelter preference, and behavioural assessments concerning light and claw banding. For this reason, this study examines the welfare implications of common aquarium practices for three species often kept in aquaria in Europe, for which more species-specific welfare-related evidence can be found: the European spiny lobster (*Palinurus elephas*), the European lobster (*Homarus gammarus*), and the American lobster (*Homarus americanus*). By exploring the existing literature on the subject, the aim of the review is to assess whether these practices result in poor welfare and to identify conditions that meet their ethological needs.

According to the Five Freedoms framework of the World Organisation for Animal Health (OIE) [11], animal welfare should be ensured by addressing the following conditions:

(1) Freedom from hunger, malnutrition, and thirst;
(2) Freedom from fear and distress;
(3) Freedom from heat stress or physical discomfort;
(4) Freedom from pain, injury, and disease;
(5) Freedom to express normal behavioural patterns.

Therefore, this review will focus on key factors such as stocking density, light exposure, shelter availability, claw banding, and water parameters, all in the context of these principles.

## 2. General Description and Biology

### 2.1. European Spiny Lobster (P. elephas)

Spiny lobsters are not true lobsters but belong to the family *Palinuridae*. Other common names are crayfish, sea crayfish, or crawfish. They are medium- to large-sized crustaceans characterised by a robust, tubular body and an absence of claws. They are found in tropical, subtropical, and temperate marine environments, preferring the rocky or muddy seabed in shallow to deep waters of up to 700 m [12]. Their typical size range is 21–60 cm [12]. The European spiny lobster, *P. elephas*, is distributed in the eastern Atlantic Ocean, from Norway to Morocco, and throughout the Mediterranean, in the Canary Islands and the Azores [13], preferring rocky substrates with numerous small caves for shelter [14]. They are typically found in water temperatures between 13 and 18 °C, with mortalities reported at temperatures above 24 °C [15], and exhibit a salinity preference of 30–40 psu [12]. They are nocturnal foragers, with more than 90% of adults returning to their shelter in the light period [16]. Nocturnal movements are believed to be linked to the moon phases, as European spiny lobster catches are reduced during the full moon, while they are significantly higher during the new moon, signifying increased activity in dull light and suggesting a strong circadian rhythm [17]. They exhibit omnivorous feeding behaviour, consuming molluscs, sea urchins, and a variety of other benthic organisms [17].

### 2.2. European Lobster (H. gammarus) and American Lobster (H. americanus)

True lobsters from the family Nephropidae, notably the European lobster (*H. gammarus*) and the American lobster (*H. americanus*), differ significantly from spiny lobsters. Both species possess two large claws, which they use for hunting and defence [18]. They are animals of the open coasts that are found in waters having a salinity of 32‰ or more [8]. European lobsters inhabit waters typically ranging from about 6 °C to 21 °C, with a preferential temperature at around 12 °C, whilst American lobsters can be found at temperatures from −1 °C to about 30 °C [19,20]. The size range is 23–50 cm for European lobster [21] and 25–64 cm for American lobster [22]. They are carnivorous foragers, consuming a variety of marine animals, including other crustaceans, molluscs, worms, starfish, and fish [8]. They inhabit the rocky seabed and are solitary creatures that require shelters for protection [8]. These lobsters are also primarily nocturnal, emerging at night to forage [8]. Their aggressive territorial behaviour is significant, with males competing for territory, shelter, and mating opportunities [23].

## 3. Environmental Enrichment

Environmental enrichment has been defined as “an animal behaviour principle that seeks to enhance the quality of captive animal care by identifying and providing the environmental stimuli necessary for optimal psychological and physiological well-being” [24] and “a deliberate increase in environmental complexity with the aim to reduce maladaptive and aberrant traits” [25]. Young [26] defined the goals of environmental enrichment practices as increasing behavioural diversity, reducing abnormal behaviour, widening the range of normal behavioural patterns, enhancing the positive use of the environment, and enhancing the ability to handle challenges.

Environmental enrichment represents a welfare-enhancing method that addresses the three main welfare concepts (feeling-based, function-based, and nature-based) [27]. The most commonly used types of environmental enrichment are [26] as follows:

(a) Physical enrichment: involving the addition of objects or substrates or alterations to the enclosure, thereby increasing environmental complexity;
(b) Sensory enrichment: providing stimuli that engage the senses, such as light, sound, or tactile elements;
(c) Dietary/nutritional enrichment: relating to the variety and delivery of food, which may involve novel or diverse types, or adjustments in the method or frequency of presentation [28];
(d) Social enrichment: involving the addition or removal of direct or indirect interactions with conspecifics or humans;
(e) Occupational enrichment: aimed at increasing territorial variability to reduce psychological and physical monotony. This can include tools that challenge animals or encourage physical activity [28].

It is particularly well-suited in barren tanks, where the environment can be adjusted and tailored with different substrate types, tank covers, wall and floor colours, lighting, objects, and tools. Environmental enrichment strategies also involve the use of items such as shelters for refuge, features that reduce aggression, and tools for cognitive stimulation. Additionally, increasing environmental complexity and variation over time boosts unpredictability and variety. Further examples for aquatic species include substrates for tank floors, incubation of eggs, and living periphyton [25,29].

By reviewing 147 studies covering 82 aquatic species, Zhang et al. [30] concluded that physically enriched environments lead to significantly improved welfare compared to barren environments, including positive impacts on the physiology, survival, health, and overall welfare. Specific welfare metrics used in these studies include increased survival rates, reduced levels of stress biomarkers (e.g., cortisol or lactate), lower aggression, enhanced growth, and more natural behaviour patterns. For example, in crustaceans, environmental enrichment such as shelters or complex substrates has been shown to reduce agonistic interactions, promote hiding behaviour, and lower injury rates. In some cases, enriched environments also resulted in improved immune responses and reduced mortality during moulting. However, such enrichment may increase labour demands due to added maintenance and cleaning [31], which is a relevant concern in holding tanks used in supermarkets, fishmongers, and restaurants, where poor hygiene could pose additional welfare risks for the animals.

The type of environmental enrichment most suited for spiny lobsters and true lobsters held in aquaria would most likely consist of the use of structures that these animals can use as shelters. As highlighted in the following section, there is a substantial literature that shows how these animals, if given the choice, prefer to spend most of their time sheltered in dark areas, away from direct light. The use of plastic films displaying a nature-based bottom pattern, such as a rocky seafloor, placed at the bottom of the tanks could mimic their natural environment and thus constitute a positive enrichment practice. Whilst this last approach might be beneficial also in terms of not increasing husbandry time, there are no studies that have investigated its effects on the wellbeing of spiny lobsters and true lobsters.

## 4. Stocking Conditions

### 4.1. Stocking Density

#### 4.1.1. Spiny Lobsters

Spiny lobsters, unlike true lobsters, are not purely solitary animals, and can be gregarious [32,33,34,35,36]. They can tolerate higher stocking densities than true lobsters, with species such as tropical rock lobsters (*Panulirus ornatus*) growing well at densities as high as 80 individuals per 16 m^2^ marine cage [37]. When reared in raceway facilities, tropical rock lobsters can tolerate densities of 14 to 43 kg/m^2^ without compromising survival rates [38].

By contrast, mud spiny lobsters (*Panulirus polyphagus*) reared in artificial ponds at high stocking densities exhibited a lower growth rate, with a lower stocking density resulting in greater survival [39]. A study on an unidentified *Panulirus* sp. showed that growth decreased at high stocking densities, with an optimal stocking density at 18 individuals per cubic metre [40]. With southern rock lobsters (*Jasus edwardsii*), juvenile growth decreased with increasing density, and the presence of shelter increased survival [41]. A study on the farming of spiny lobster *Panulirus* sp. in floating net cages found better growth and survival at 10 individuals per cubic metre compared to 15, 20, or 25 per cubic metre [42]. Sakthivel et al. [43] monitored *Panulirus homarus* at densities of 5, 10, 15, 20, and 25 individuals in 120 L aquaria and noted that those reared at 15 individuals per aquarium exhibited better food intake and growth, whilst those reared at 25 individuals per aquarium exhibited poorer food intake and growth. In another study, the highest growth rate achieved in indoor culture of the juvenile spiny lobsters *P. homarus* was at a density of seven individuals/m^2^ [44].

It must be noted that the available literature on spiny lobsters, particularly *P. elephas*, is predominantly focused on production traits such as survival and growth in captivity, with limited emphasis on behavioural studies that address welfare needs. While survival and growth are important indicators of overall health, they do not provide a comprehensive understanding of the behavioural requirements in captive environments. Further research is needed to assess the impact of stocking density on the welfare of these animals, with investigations going beyond only mortality and including behavioural signals (tail-flipping as an escape response, limb autotomy as a sign of extreme distress, reduced or absent limb movement, no reaction when body is touched or moved, no attempt to move when placed on the side, changes in social interactions such as increased instance of agonistic behaviours), physical signs (distorted membranes between the tail and carapace or at leg joints, observable injuries or physical damage), physiological changes (elevated L-lactate levels in the haemolymph), and suppressed growth or feeding (less practical in short-term assessments).

#### 4.1.2. True Lobsters

Both American and European lobsters are solitary [7,8], and aggressive competition for shelter and territory is common [8]. They usually maintain individual shelters [45], presumably to obtain protection from predators [7]. Lobsters readily fight and maintain stable dominance hierarchies in captivity [46]. In particular, males fight for shelter, but only the locally dominant male will secure an undisturbed shelter, which may lead to mating with multiple females [47].

In general, when a group of lobsters is introduced to a new tank, physical fighting occurs [48]. This results in the victory of various individuals (dominant) and the defeat of others (subordinates), and the lobsters establish themselves in a relatively stable hierarchy. Interactions become simple approach and retreat events, with the subordinate lobsters generally avoiding the approach of the dominants [49]. Karnofsky & Price [45] created a semi-natural aquatic habitat for lobsters in a 180 m^2^ pool. This large tank housed thirty American lobsters, which were studied to determine the relationship between social dominance, judged by approach–withdrawal sequences, and the use of space. It was noted that the frequency of high-level aggressive acts was much lower than in previous studies in smaller tanks. Large males initiated agonistic interactions much more frequently than other categories, in particular directing their attention towards other large males and large females. Individuals were more likely to win interactions with otherwise dominant individuals if they defended their shelter, suggesting a shelter-based form of territoriality. Furthermore, dominant individuals often kept the shelters immediately adjacent to their own shelter free of other lobsters.

### 4.2. Light

Exposure to bright light can adversely affect decapod crustaceans [50], leading to irreversible damage to their photoreceptors [51]. However, a complicating factor in both qualitative and quantitative studies on the vision of crustaceans is the variation in structural and functional responses of their eyes, which depend on the time of day and the individual’s prior exposure to light [51]. For example, the long-term negative effects of bright light on the structural integrity and performance of spiny lobster eyes largely depends on the intensity of ambient light and the state of light adaptation prior to the experiment [51]. Specifically, southern rock lobster (*J. edwardsii*) kept under normal 12 h dark/light conditions and exposed to white light of approximately 60,000 lux for roughly 3 s, showed damage to cones and retina one week after. Another specimen, after being exposed to sunlight for 7 h over a period of one week, displayed damage to dioptric structures and the retina. Another specimen, after being exposed to sunlight for 4 h on the first day, 4 h on the second day, 30 min on day 35, and 150 min on day 48, showed severe eye damage [51].

Circadian rhythms, which regulate sleep–wake cycles and activity patterns, influence the behaviour and welfare of decapod crustaceans [52]. Both spiny lobsters and clawed species exhibit circadian-controlled activity patterns, typically being more active during the night [53,54]. Exposure to constant or inappropriate lighting conditions can disrupt these rhythms, potentially leading to abnormal behaviour, stress, and reduced welfare.

### 4.3. Shelters

#### 4.3.1. Spiny Lobsters

European spiny lobsters in the wild prefer habitats with abundant shelters, such as holes and crevices, for refuge. In captivity, these animals have shown a clear preference for certain shelter shapes and sizes. For example, 55% of young European spiny lobsters in captivity selected semi-circular shelter, while only 16.5% selected square ones and 11% chose circular shelters [55]. There was a positive linear relationship between preferred shelter diameter and carapace length.

A study comparing the effects of different shelter shapes made from polyvinyl chloride (PVC) on the physiological response and growth of the scalloped spiny lobster, *P. homarus*, demonstrated the benefits of individual shelters. These shelters helped reduce contact between individuals, thus decreasing the likelihood of cannibalism. Among the various shapes tested, the square shelter resulted in lower stress levels, as indicated by reduced glucose and haemolymph protein levels, as well as better growth [56]. Other studies have also shown that shelters suspended in the water column increased survival rates for several spiny lobster species [57,58,59].

#### 4.3.2. True Lobsters

Studies on true lobsters note the importance of shelters and hiding behaviour [8,60,61]. The preference for unlit areas was highlighted in a study in which European lobsters (*H. gammarus*) were presented with a maze during periods when the lights were on or off. Five minutes after the lights went out, the lobsters left their shelters and began wandering through the maze; during the light period, however, the lobsters remained in their shelters and only left them occasionally [60]. In a study in which American lobsters (*H. americanus*) were monitored for three years in their natural environment, it was observed that these animals spent most of their time hidden in shelters under algae, rocks, and boulders, only coming out at night. Some frequently changed shelters, whereas others remained in the same shelters for up to ten weeks [23]. Similar observations were made by Ennis [62], who reported that American lobsters spent the majority of their time in shelters, leaving them only at night, and that each shelter was typically occupied by only one individual. O’Neill & Cobb [63] also noted that in laboratory conditions, it was rare for more than one lobster to occupy a shelter. Additionally, Paille et al. [64] found that American lobsters kept in tanks with shelters spent over 95% of their time inside the shelters.

American lobsters were observed in both natural and laboratory settings, where they showed a preference for low-profile shelters, with a height less than the width. This preference was confirmed when they were offered the choice between low-profile (height = 1/2 width) and high-profile (height = width) openings. Moreover, given the choice between transparent or opaque shelters, American lobsters preferred the opaque ones [65].

A study on environmental enrichment in aquaria housing American lobsters (*H. americanus)* showed how increased habitat complexity reduced the intensity of fighting. The authors concluded that adding shelters and environmental enrichment could be a cost-effective way to reduce aggression in lobsters, thereby supporting their ethological needs and wellbeing [66].

Van der Meeren [67] examined the behaviour of hatchery-reared European lobsters (*H. gammarus*) when shelters were available. Upon introduction to a new sand tank, the lobsters immediately sought shelter, either hiding under it, or burying themselves in the sand next to it. This behaviour occurred even in animals that had never encountered shelters before.

These studies strongly support the idea that both spiny lobsters and true lobsters naturally spend the majority of their time in shelters, often in near-complete darkness. This behaviour is also observed in other decapod crustaceans. For example, studies on the common shore crab (*Carcinus maenas*), striped coastal crab (*Pachygrapsus crassipes*), and Louisiana red crayfish (*Procambarus clarkii*) revealed that they also prefer dark shelters over bright open areas [68,69,70].

### 4.4. Claw Banding

In captivity, true lobsters usually have strong elastic bands placed over the pincers to prevent their opening. This is used to prevent mutual harm between individuals and ensure operator safety and might seem beneficial for animal welfare by protecting them from injuries and aggression. However, restricting both claws can be highly detrimental to lobsters, as they are used for feeding, defence, and even locomotion [8]. Furthermore, prolonged banding can cause muscle atrophy, impair natural feeding, and hinder defensive actions, and may weaken the claws during moulting [8,10]. These limitations severely restrict the ability to exhibit normal behaviour, as argued by several authors [8,10,71]. While claw banding may reduce injuries, the practice is incompatible with the biological and behavioural needs of lobsters, and by restricting vital behavioural functions it might significantly undermine their overall welfare. Allowing lobsters to express natural behaviour is crucial for maintaining both their physiological health and welfare in captivity [71].

It is important to note, however, that most of the evidence on the negative impacts of claw banding concerns long-term effects, even though lobsters are typically held in captivity for relatively short periods, particularly in commercial settings such as restaurants.

### 4.5. Water Parameters

Dissolved oxygen (DO), pH, temperature, and chemical contaminants can exert a profound influence on the welfare, physiology, and overall viability of spiny lobsters and lobsters in captive systems. Low DO is associated with a multitude of stress responses, including increased heart rate, altered haemolymph chemistry, and a shift towards anaerobic metabolism [72]. Chronic hypoxia induces physiological dysregulation, making lobsters susceptible to opportunistic pathogens and parasites [73]. The pH of the water affects numerous biochemical and physiological reactions. Despite compensation for modest fluctuations, chronic or extreme deviations in environmental pH can lead to impaired enzymatic activity and diminished oxygen-carrying capacity of the haemolymph [74]. Each species has an optimal thermal window, often shaped by its native habitat. However, these three species can adapt to a relatively wide thermal range, given their diverse distribution (see Section 2.1). Ammonia (NH_3_) is a principal metabolic by-product and a common pollutant in captive aquatic systems. Through nitrification, ammonia is converted to nitrite (NO_2_) and subsequently to nitrate (NO_3_), each less toxic than the previous. Trace metals such as copper, cadmium, and zinc can accumulate in crustacean tissues, especially under conditions of low pH that promote metal solubility [75]. Although lobsters have some capacity for detoxification via haemolymph binding proteins, chronic exposure can lead to immunosuppression and hepatopancreatic lesions [74].

#### 4.5.1. Spiny Lobster

A breakdown with numeric thresholds for each parameter rarely exists in the primary literature for the European spiny lobster. General preferences can, however, be inferred from the natural habitat, reported in Section 2.1, with a suitable temperature range between 13 and 18 °C and a salinity preference of 30–40 psu. Given the scarcity of other water quality parameters in the literature, the conditions can be inferred from data for closely related species, such as the scalloped lobster (*P. homarus*). The optimal conditions of dissolved oxygen to support *P. homarus* in aquaculture range from 2.7 to 5.4 mg/L [75], with a nitrite concentration of less than 5 mg/L [76]. For the southern rock lobster (*J. edwardsii*), held in recirculation systems, the nitrate concentrations should be <100 mg/L [75]. For *Palunirus* sp., Thesiana et al. [77] report the water quality requirement for spiny lobster aquaculture to be DO > 4 mg/L and ammonia (NH_3_) less than 0.1 mg/L. Data on the tolerance of nitrite cannot be found in the literature for this genus, although it can be inferred that it is likely to be similar to the values for *Homarus* spp., given the similarity in values for ammonia and nitrate tolerance.

#### 4.5.2. True Lobsters

Van Olst et al. [78] reviewed water quality parameters for *Homarus* species and noted that, apart from temperature (most suitable range 18–22 °C), optimal conditions generally align with their natural environment, such as a pH between 7.8 and 8.2, although they can withstand considerable environmental fluctuations [79]. Whiteley et al. [80] found that lobsters can tolerate dissolved oxygen levels as low as 0.2 mg/L in 5 °C seawater (33‰) and 1.72 mg/L in 25 °C brackish water (20‰). However, oxygen supersaturation can be harmful, as it may cause gas bubbles to form in the haemolymph, restricting blood flow [81]. While lobsters can endure fluctuations in salinity, optimal levels range between 28 and 35‰ [78,82,83], meaning that some freshwater dilution, such as during filter flushing, is feasible. Among water quality parameters, ammonia is likely the most critical limiting factor in recirculating seawater systems, with van Olst et al. [78] proposing a higher acceptable concentration than the <1.5 mg/L recommended by D’Abramo et al. [83]. Estrella [84] suggested that short-term exposure to nitrite levels up to 5 mg/L and nitrate levels up to 100 mg/L may be tolerable.

Effective ammonia management in commercial lobster operations relies on maintaining high water quality through biological filtration that converts toxic ammonia into less harmful compounds, combined with mechanical filtration to remove solids. Regular water exchange and aeration, controlled feeding to prevent excess waste, and proper stocking densities help minimise ammonia buildup. Maintaining optimal pH and temperature is also critical, as these factors influence ammonia toxicity and filtration efficiency.

A summary of key distinctive features and suitable water quality conditions for spiny lobsters and lobsters is given in Table 1.

## 5. Recommendations

There are significant differences in the stocking density requirements for spiny lobsters and true lobsters, as the former can be gregarious, while the latter are solitary and highly territorial. Spiny lobsters can tolerate relatively high stocking densities, although tolerating does not necessarily translate into adequate conditions of wellbeing that satisfy the ethological needs of the animals. For true lobsters, it is likely that being in close proximity to a number of individuals causes them significant stress.

In their natural environment, both spiny lobsters and true lobsters prefer to take refuge in crevices or under rocks and boulders, spending most of their time sheltered in almost complete darkness. This is confirmed in controlled studies by their behaviour towards artificial shelters, which they utilise extensively. These needs should be met in captivity. Yeap et al. [85] suggest that adequate shelters, of the correct shape and size, should be provided in crustacean holding environments to facilitate the natural behaviour and reduce potential stress. Additionally, sufficient space should be allocated for foraging [85]. According to Eggleston & Lipcius [86], shelters made from materials such as plastic or terracotta pipes can help meet these needs.

Spiny lobsters and true lobsters are primarily nocturnal and emerge from their shelters during the night to forage for food, returning to shelter as light levels increase [8] and remaining there, emerging only occasionally during periods of light [60]. This behaviour highlights their need to avoid bright light. Beard & McGregor [8] recommend that lobsters kept in captivity should not be exposed to strong lighting, and sudden increases in light levels should be avoided. Similarly, Jacklin & Combes [87] recommend that live decapod crustaceans should always have access to shelters and dark areas.

Claw banding is a practice that does not account for the physiological and ethological needs of these animals. While there are practical reasons for immobilising lobsters, such as avoiding injuries between the animals themselves and the operators, there are alternative methods that can avoid these problems and at the same time maintain the full use of claws. A shift towards alternative methods, such as improving environmental conditions, providing more space, and increasing shelter provision, might reduce aggressive interactions, making such restrictive measures redundant; installing partitions within the aquarium would then obviate the need for claw banding and effectively eliminate any risk of aggression. In fact, using simple, cost-effective aquarium separators can prevent cannibalism and/or aggression. To avoid the risk of injury to operators, tools such as pliers with rubber tips can be used to handle the lobsters without damaging their carapace. Inadequate handling and maintenance can lead to high mortality rates in several crustacean species [84]. Similar to how nets are routinely used to handle aquatic animals such as fish, similar methods should also be adopted for crustaceans.

Daily or every other day measurements of DO, temperature, pH, and ammonia should be supplemented with regular testing of nitrite, nitrate, and key metals. Automation of these measurements via sensors and digital controllers can reduce human error and facilitate real-time adjustments [72]. Effective mechanical filtration, combined with aquarium biofiltration systems, is indispensable in maintaining low ammonia and nitrite concentrations. Enhancing aeration, through diffusers, helps sustain DO above 6 mg/L. Flow rates should be calibrated to maintain stable temperature gradients and uniform distribution of oxygen and dissolved nutrients. Maintaining species-specific temperature ranges can be achieved through the use of heat exchangers or chillers, and temperature fluctuations should be minimised. To avoid the risk of toxic conditions for the animals, water should be filtered and devoid of heavy metals and other contaminants. Implementing routine behavioural observations augments traditional water chemistry assessments, granting a more holistic perspective on lobster welfare [75]. Maintaining appropriate pH, dissolved oxygen, temperature, and low levels of chemical impurities is fundamental to safeguarding the welfare of captive spiny lobsters and true lobsters. The tight interdependencies among these parameters underscore the necessity for rigorous monitoring, robust filtration, and proactive management practices. By adhering to established guidelines, operators can minimise physiological stress and bolster immune competence for these animals.

Based on the evidence, conditions for keeping spiny lobsters and true lobsters should meet the following criteria: no claws should be tied (for true lobsters), each animal should have sufficient space to avoid direct contact with others, the aquarium should have very dim light or be kept in complete darkness, and each animal should have a shelter large enough for its entire body. Additionally, since true lobsters do not naturally cohabit, they should be kept separately.

**Figure 1:**
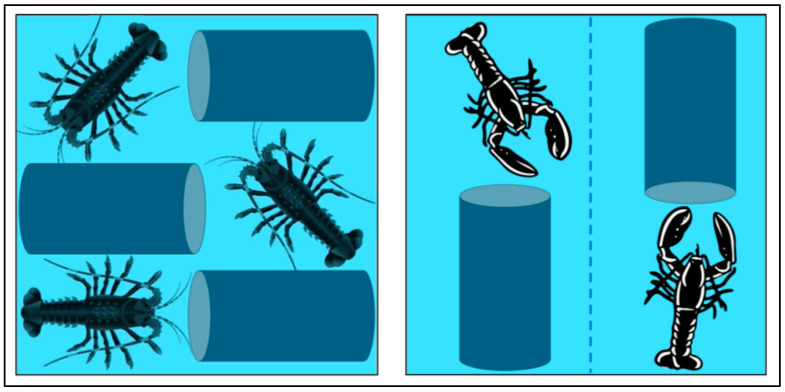
Representation (top view) of spiny lobsters (**left**) and true lobsters (**right**) held in a one-metre square tank, showing the maximum number of individuals that can be kept without compromising their freedom of movement [88,89]. Tanks include PVC pipes to be used as shelters for both groups of animals, and a divider (dotted line) in the middle of the tank for true lobsters, which are kept with their claws untied.

The following recommendations are proposed for the management of spiny lobsters and true lobsters in aquaria: spiny lobsters and true lobsters should be housed in separate aquaria, with each tank containing only spiny lobsters or only true lobsters; for spiny lobsters, the density should not exceed five individuals per square metre, and each animal should have sufficient space to avoid contact with other individuals (Figure 1). This should align with their natural behaviour as outlined in Section 2, as well as with the concept of the Five Freedoms; true lobsters should never have their claws tied; aquaria must be equipped with separators that divide the aquarium into different areas (Figure 1), with only one animal per separate area; the compartments should be large enough for the animal to turn around, thus resulting in five animals per square metre as the maximum stocking density allowed; both spiny lobsters and true lobsters must have access to shelters, such as PVC pipes, which provide adequate space for hiding. Each animal should have its own shelter, which must be at least as long as the animal’s body and kept clean and free from organic buildup and debris (Figure 1); no direct light should be allowed above the aquaria; water quality parameters should align with those laid in Table 1. These parameters should be checked and maintained within suitable ranges, as laid out in Section 4.5.

## 6. Considerations

These measures represent a significant improvement to the typical conditions for keeping these animals, ensuring their ethological and physiological needs are met. This includes protection from direct light, access to shelters, prevention of direct contact with other individuals, suitable water quality parameters, and for true lobsters, their claws untied. However, the relationship between stocking density and welfare is complex, as evidenced by research across various animal taxa, including poultry and fish. Studies in these animal groups have demonstrated that while higher stocking densities can negatively impact welfare due to factors like stress, aggression, and resource competition, the precise effects often depend on a range of interacting variables, including environmental enrichment, resource availability, and species-specific behaviour [90,91].

The stocking density recommended for spiny lobsters and true lobsters is based on the precautionary principle, ensuring that the animals have enough space to move around without touching each other. This recommendation is made in the absence of studies directly investigating the effects of stocking density on their wellbeing. Further research is needed to better understand how stocking density interacts with other environmental factors and impact welfare in these species.

Considering all relevant factors, it would be ideal if these animals, which appear to be sentient and capable of experiencing pain and suffering [6], were not sold alive in supermarkets, restaurants, or fishmongers. The conditions in commercial establishments are in fact often insufficient to meet their needs. The practice of keeping these animals in aquaria is largely driven by cultural norms and the widespread misconception that these conditions help preserve their “freshness”. This belief, coupled with the assumption that selling them dead (whether refrigerated or frozen), would negatively affect their taste, perpetuates the use of holding tanks for live animals. In this regard, some countries, such as Switzerland, have already taken significant steps to curtail the practice of selling live decapod crustaceans, with legislation prohibiting the sale of these animals alive to consumers [92]. Implementing similar bans in more countries would prevent these animals from being kept in aquaria under conditions that are unlikely to meet their welfare needs. In the United Kingdom, several major retailers have voluntarily suspended sales of live decapods because of welfare concerns [2]. By banning the sale of live animals to the general public, governments could ensure that welfare standards are upheld, promoting more humane treatment and reducing the stress and harm inflicted on these species.

## 7. Conclusions

In light of the scientific evidence, ethical considerations, and emerging policy changes, it is increasingly clear that current practices for keeping live decapod crustaceans in commercial settings fall short of meeting their welfare needs. While incremental improvements, such as shelter provision, appropriate lighting, and improved water quality, can mitigate some of the stressors these animals face, they are unlikely to fully address the welfare implications of live storage. A precautionary and ethically responsible approach would be to eliminate the sale of live decapods to consumers. The aim should be to align commercial practices with animal welfare science and public ethical standards.

## Figures and Tables

**Table 1:** Desirable levels for key water quality parameters for spiny lobsters and true lobsters.

Parameters	Spiny Lobsters	True Lobsters
Distinctive physical features	Absence of claws	Two large claws
Size range (cm)	21–60	25–64
Diet	Omnivorous (molluscs, sea urchins, and other benthic organisms)	Omnivorous (crustaceans, molluscs, worms, starfish and fish)
Feeding behaviour	Nocturnal foragers	Nocturnal foragers
Social behaviour	Solitary or gregarious	Solitary and territorial
Shelter preference	Semi-circular	Height less than width, opaque
Enrichment needs	Shelters	Shelters
Light regime	Full darkness	Full darkness
Temperature (°C)	13–18 *	18–22
Salinity (‰)	30–40 *	28–35
DO (mg/L)	2.7–5.4	6.4
pH	7.8–8.2 **	7.8–8.2
Ammonia (mg/L)	<0.1	<0.14
Nitrite (mg/L)	<5 *	<5
Nitrate (mg/L)	<100	<100

* Based on natural conditions (Section 2.1). ** Scarcity of data, but likely similar to true lobsters.

## Data Availability

No new data were created or analyzed in this study.
